# Evaluation of bioactive secondary metabolites from endophytic fungus *Pestalotiopsis neglecta* BAB-5510 isolated from leaves of *Cupressus torulosa* D.Don

**DOI:** 10.1007/s13205-016-0518-3

**Published:** 2016-09-29

**Authors:** Deeksha Sharma, Avijit Pramanik, Pavan Kumar Agrawal

**Affiliations:** 1 Department of Biotechnology, G. B. Pant Engineering College, Ghurdauri, Pauri, Uttarakhand, 246194 India; 2 Department of Microbiology, Central University of Haryana, Mahendergarh, Haryana 123031 India

**Keywords:** *Pestalotiopsis neglecta*, Antibacterial activity, Cytotoxic assay, GC-MS analysis, Bioactive metabolites

## Abstract

Six endophytic fungi were isolated from *Cupressus torulosa* D.Don and identified phenotypically and genotypically. The fungal cultures were further grown and the culture was extracted by two organic solvents methanol and ethyl acetate. The screening was carried out using the agar well diffusion method against human pathogen such as *Escherichia coli*, *Salmonella typhimurium, Bacillus subtilis* and *Staphylococcus aureus.* Isolated strain of *Pestalotiopsis* sp. was showing prominent antibacterial activity. The crude methanol and ethyl acetate extract of *Pestalotiopsis* sp. showed MIC of 6.25 mg/mL for *S. typhimurium and S. aureus* which showed its efficacy as a potent antimicrobial. The phytochemical screening revealed the existence of a diverse group of secondary metabolites in the crude extracts of the endophytic fungi that resembled those in the host plant extracts. On the basis of phenotypic characteristics and rDNA sequencing of the ITS region of the endophyte was identified as *P. neglecta* which turned out to be a promising source of bioactive compounds. There is little known about endophytes from *C. torulosa* D.Don. In this paper we studied in detail the identification of isolated endophytic fungi *P. neglecta* from *C. torulosa* D.Don and characterization of its active metabolite compounds. The partially purified second fraction (PPF) extracted from the fungal culture supernatant was subjected to gas chromatography followed by mass spectrometry which revealed the presence of many phytochemicals. These results indicate that endophytic fungi *P. neglecta* isolated from medicinal plants could be a potential source for bioactive compounds and may find potential use in pharmaceutical industry.

## Introduction

An increase in the number of people in the world having health problems caused by various cancers, drug-resistant bacteria, parasitic protozoans, and fungi is a cause for alarm (Strobel 2003). Development of multiple drug-resistant microbes raised the need to search for new and novel antimicrobials for treatment of human diseases (Wise 2008). An intensive search for newer and more effective agents to deal with these disease problems is now under way and endophytes are a novel source of potentially useful medicinal compounds. Much of microbial diversity of nature remains to be explored, particularly marine microbial environments (Newman et al. 2003). Microorganisms have the ability to utilize various substrates as a consequence of the diversity of their biological and biochemical evolution (Fernandes et al. 2009). The solid substrates they use include, among others, live plants. Both bacteria and fungi are known to cooperate with many plants to form mutually beneficial associations. Actinomycetes and fungi, of all microorganisms studied, have been found to be the most prolific producers of secondary metabolites (Guanatilaka 2006).

There are enormous scopes exist for the recovery of novel fungal species, genera and biotypes from this ecological niche. To some estimate approximately 1.5 million fungal species exist in the world (Hawksworth 1991; Hawksworth 2001) while only 100,000 have been discovered and there may be at least one million species of endophytic fungi alone (Dreyfuss and Chapela 1994). In the last few years, considerable knowledge has been accumulated on the biology of endophytic microorganisms (Firakova et al. 2007). Endophytes comprise a large but little explored share of fungal diversity (Yuanab et al. 2011; Perottoab et al. 2013).

A range of microbial species are known to be endophytic, colonizing inter and intracellular spaces of tissues of higher plants without causing apparent damage on the plants in which they live. Often they have proven to be rich sources of bioactive natural products (Li et al. 2008; Molina et al. 2012). Mutualistic interactions between endophytes and host plants may result in fitness benefits for both partners (Kogel et al. 2006). The endophytes may provide protection and survival conditions to their host plant by producing a plethora of substances which, once isolated and characterized, may also have potential for use in industry, agriculture, and medicine (Porras-Alfaro and Bayman 2011).

Almost all the plant species (300,000) growing in unexplored area on the earth are host to one or more endophytic organisms (Strobel and Daisy 2003). To date, only a few plants are investigated for their endophytic biodiversity and their potential to produce bioactive secondary metabolites. Studies have been conducted at different parts of the world about the endophytic biodiversity, taxonomy, reproduction, host ecology and their effect on host (Bandra et al. 2001). Currently, endophytes are viewed as outstanding sources of bioactive natural products, because many of them are occupying literally millions of unique biological niches growing in so many unusual environments.

Endophytic fungi are of biotechnological interest due to their potential as a source of secondary metabolites that has been proven useful for novel drug discovery (Yan et al. 2011). Antifungal and antibacterial activities of plant endophytic fungi have been reported by several groups (Liang et al. 2012; Gherbawy and Gashgari 2014; Idris et al. 2013; Bhardwaj et al. 2015). Endophytic fungi has been shown to produce several pharmacologically important compounds such as antimycotics steroid 22-triene-3β-ol (Metwaly et al. 2014), anticancer cajanol (Zhao et al. 2013), podophyllotoxin and kaempferol (Huang et al. 2014), anti-inflammatory ergoflavin (Deshmukh et al. 2009), antioxidant lectin (Sadananda et al. 2014), insecticidal heptelidic acid (Zhang et al. 2014), immunosuppressive sydoxanthone A, B (Song et al. 2013) and cytotoxic radicicol (Turbyville et al. 2006).

Metabolites produced by endophytes are being recognized as a versatile arsenal of antimicrobial agents. Some endophytes have been known to possess superior biosynthetic capabilities, owing to their presumable gene recombination with the host, while residing and reproducing inside the healthy plant tissues (Li et al. 2005). A high proportion of endophytic fungi (80 %) produce biologically active compounds in tests for antibacterial, fungicidal and herbicidal activities (Schulz et al. 2002). The continued development of new antimicrobial compounds is important to overcome the difficulties related to the treatment of infections caused by resistant pathogens in accordance with Petersen et al. (2004). Thus, it can be said that endophytic fungi have emerged as an alternative source for the production of new antimicrobial agents.

Plants used in traditional medicine have played a very important role in the search for new bioactive strains of endophytic fungi, as it is possible that their beneficial characteristics are a result of the metabolites produced by their endophytic community (Kaul et al. 2012; Kusari et al. 2013). As higher plants are known to harbor endophytic fungi (Bhardwaj et al. 2015) that are believed to be associated with the production of pharmaceutically important products, in this context, the aims of this work were to characterize the fungal endophyte *Pestalotiopsis* sp. BAB-5510 associated with *Cupressus torulosa* D.Don from Pauri, Garhwal region of Uttarakhand and to detect cytotoxic and antimicrobial activities of these fungi against some pathogenic microbes.

Despite this potential, a repertoire of medicinal plants remains to be studied regarding their endophytic composition, for example *C. torulosa* D.Don. This is a well-known medicinal plant whose leaves have been proven to have anti-inflammatory, anticonvulsant, antimicrobial, and wound-healing properties (Leite et al. 2004, 2006; Carli et al. 2010; Luiz-Ferreira et al. 2011; Almeida et al. 2013; Chen et al. 2013; Bezerra dos Santos et al. 2015). Due to the medicinal properties of *C. torulosa*, this species was the focus in the present study for a search of endophytic fungi that is able to produce bioactive substances with antimicrobial activity and cytotoxic activity.

Due to the importance of secondary metabolite production by endophytic fungi, the study of these *C. torulosa* associated fungi provides greater understanding of its diversity. This study is the first report about the antimicrobial and cytotoxic activity of endophytic fungi residing in *C. torulosa* leaves in which the fungus *Pestalotiopsis neglecta* demonstrated the ability to produce bioactive agents with pharmaceutical potential, and may provide a new lead in the pursuit of new biological source of drug candidates.

## Materials and methods

### Sample collection and isolation of endophytic fungi

The sampling scheme was designed with the intention of isolating endophytic fungi from mature and healthy needle of *C. torulosa* D.Don (family: C*upressaceae*) from Pauri, Garhwal region. The plant samples were tightly sealed in polythene bags under humid conditions and kept at room temperature. The voucher specimen was deposited at Botanical Survey of India, Dehradun with accession number 115744. The isolation of the fungal endophytes commenced within 24 h of collection of plant samples.

Needles were cut into 5 mm long segments. Surface sterilization was done by following the method described by Arnold et al. (2007) with minor modification. Needle segments were surface sterilized by consecutive immersion for 1 min in 75 % Ethanol then for 1 min in 0.1 % mercuric chloride. Surface sterilization was followed by several washing steps in autoclaved distilled water (Bisht et al. 2016). The time of the dilution and immersion in ethanol and Mercuric chloride varies with tissues and host (at least three washing require). Under sterile conditions, tissue segments were allowed to surface-dry before plating (Petrini et al. 1982). Five needles segments were then evenly placed in PDA plates augmented with 50 μg/mL of chloramphenicol to avoid bacterial contamination. Plates were sealed with parafilm and incubated at 27 ± 2 °C for 5–8 days in BOD incubator. Hyphal tips of the developing fungal colonies were transferred aseptically to fresh PDA plates to get pure cultures of the growing fungi.

### Phenotypic and genotypic identification of the endophytic fungi

The isolated endophytic fungi were initially identified through microscopic examination of colony morphology and reproductive characteristics using slide cultures (Bhardwaj et al. 2014). Primary confirmation of morphological identification of the endophytic fungi was carried out at Forest Research Institute (FRI), Dehradun, India. For genotypic identification, total genomic DNA of the endophytic fungi were isolated directly from actively growing mycelium growing in potato dextrose broth (PDB), using DNA extraction kit (Genei). Fungal DNA was extracted in the laboratory using the protocol of Kariyawasam et al. (2012). The extracted DNA was subjected to the polymerase chain reaction (PCR) using primers ITS1: TCCGTAGGTGAACCTGCGG and ITS4: TCCTCCGCTTGATATGC for amplification of ITS region (White et al. 1990). Amplified DNA was subjected to DNA sequencing and this DNA sequence was compared with already existing DNA sequences in NCBI GenBank (http://www.ncbi.nlm.nih.gov.blast) to identify the respective fungi. PCR and DNA sequencing was done by the Gujarat State Biotechnology Mission, India. The acquired gene sequence was submitted to the NCBI Gen Bank database and an accession number was obtained.

### Secondary metabolite extraction

The fungi were cultivated on PDB by inoculating selected endophyte cultures in 250 mL Erlenmeyer flask containing 100 mL of the medium. The flask was incubated at 28 °C for 2 week with periodical shaking at 150 rpm. After the incubation period, the fermentation broth of the fungus was homogenized by adding 10 % methanol to it. Metabolite was extracted by solvent extraction procedure using ethyl acetate and methanol as organic solvents. To the filtrate equal volume of solvents were added, mixed well for 10 min and kept for 5 min till the two clear immiscible layers formed. The upper layer of solvent containing the extracted compounds was separated using separating funnel. Solvent was evaporated and the resultant compound was dried in rotator vacuum evaporator to yield the crude metabolite (Bhardwaj et al. 2015). The crude extract was then dissolved in Dimethyl sulphoxide at 1 mg/mL of concentration and kept at 4 °C.

### Determination of antibacterial activity of fungal crude extracts

Antibacterial activity of secondary metabolites extracted from *P. neglecta* was screened against Gram-positive and Gram-negative bacterial pathogen such as *B. subtilis, S. aureus, E. coli* and *S. typhimurium* using agar well diffusion method. Bacterial pathogens were spread on Muller Hinton agar (MHA) plates. Then wells were bore on the agar plates and three concentration of crude extract were poured in separate wells 200, 150, 100 µL. Antibacterial activities were detected after an incubation of 24–48 h at 37 °C. The presence of zone of clearance on plates was used as an indicator of bioactive nature of the strain. As positive control, streptomycin was used and DMSO was used as negative control. Three replicates were carried out for each antibacterial activity test.

### Determination of minimum inhibitory concentration

MIC was determined after antibacterial activity of the fungal crude extracts by the standard method described by Wariso and Ebong (1996) with minor modification. Muller Hinton Broth (MHB) was made and sterilized using autoclave. One milliliter of the prepared broth was dispensed into the test tubes labeled from 1 to 5 using sterile syringe and needle. A stock of   MHB containing 25 mg/mL of the crude extract was prepared. The sterile MHB with 25 mg/mL crude extract was diluted twofold for five times in sterile tubes aseptically. Then, each tube was inoculated with equal volume of overnight grown bacterial culture. Tube 6 was used as a control for sterility of the medium and tube 7 for viability of the organisms. The final concentration of the extract in each of the test tubes numbered after dilution 25, 12.5, 6.25, 3.125, 1.563 mg/mL were incubated at 37 °C for 24 h and examined for growth. The lowest dilution test tube in which growth failed to occur was the MIC of the culture.

### Phytochemical screening of fungal crude extracts

Preliminary phytochemical analysis of the crude extracts of fungi was carried out for the presence of the following metabolites: alkaloids, flavonoids, tannins, phenols, saponins, terpenoids and carbohydrates using standard methods with modification (Devi et al. 2012; Bhardwaj et al. 2015).

### MTT cytotoxic assay

This method is based on the ability of live but not dead cells to reduce a tetrazolium dye to a purple formazan product. In brief, approximately 5 × 10^3^ cells/well of HEK cell line which is a tetraploid non-malignantly transformed human embryonic kidney cell line were seeded into 96 well plates, 100 µL of High Glucose Dulbecco’s medium (HGD) was added and incubated for 24 h as to attain log phase of the cells. After 24 h, different concentrations of fungal crude extract 10, 1.0, 0.1 and 0.01 µg/mL were added into the plates and are incubated for two time points 24 and 48 h. After respective incubation period 20 µL of MTT (5 mg/mL) was added and incubated for 2 h at 37 °C in a CO_2_ incubator. After incubation medium was discarded and 200 µL of DMSO was added to dissolve the formazan crystals. Then absorbance was read in a spectrophotometer at 560 nm and cell survival was calculated by the following formula:

$${\text{Viability}}\% = {\text{Test OD}}/{\text{Control OD}} \times 100$$

### Characterization of bioactive compounds

#### Chromatographic detection and partial purification of bioactive metabolite

Thin layer chromatography (TLC) was performed on methanolic crude extracted from the culture broth of the endophyte with minor modification of Verma et al. (2014). For this, the crude fraction was spotted (50 μL) on the TLC plate and chromatography was performed by employing solvent system dichloromethane: methanol (90:10 v/v). Spots were visualized by spraying with ceric sulfate; silica residue was extracted and centrifuged and the supernatant was transferred to a microcentrifuge tube. The silica-free supernatant was checked for antibacterial activity. Preparative TLC was carried out to obtain the partial purified sample which showed antibacterial activity.

#### Detection of bioactive compounds by GC-MS analysis

The compounds separated by TLC were identified using gas chromatograph. The purified methanolic crude extract was subjected to GC-MS analysis to identify the bioactive compounds. The GC–MS-MS analysis of the crude extracts was carried out in a Shimadzu GC-MS-QP 2010 Plus fitted with a RTX-5 (60 m × 0.25 mm × 0.25 µm) capillary column in JNU, New Delhi. The instrument was set to an initial temperature of 70 °C, and maintained at this temperature for 2 min. At the end of this period the oven temperature was rose up to 280 °C, at the rate of an increase of 5 °C/min, and maintained for 9 min. Injection port temperature was ensured as 260 °C and Helium flow rate as 1 mL/min. The ionization voltage was 70 eV. The samples were injected in split mode as 10:1. Mass spectral scan range was set at 45–450 (*m/z*). The identification of bioactive compounds present in the extracts was performed by comparing the mass spectra with data from NIST05 (National Institute of Standards and Technology, US), WILEY 8, and FFNSC1.3 (Flavour and Fragrance Natural and Synthetic Compounds) libraries. The name, molecular weight and structure of the components of the test material were ascertained.

## Results and discussion

### Isolation and identification of the endophytic fungal strain

In this study, fungal endophytes associated with coniferous plant *C. torulosa* D.Don, was studied to evaluate the production of bioactive compounds. The plant was taxonomically identified and authenticated by Botanical Survey of India, Dehradun, Uttarakhand. The voucher specimen was deposited there with register number 115744. A total of six different endophytic fungi associated with leaves of *C. torulosa* D.Don were isolated, and morphotypically and genotypically identified as *Alternaria alternata*, *Daldinia* sp.*, Penicillium oxalicum* and *Pestalotiopsis* sp. (Tables 1, 2). The majority of the recovered taxa belong to the Ascomycota. This result supports the Bhardwaj et al. (2015) finding that fungal endophytes from coniferous plant *Pinus roxbrghii* mainly belong to the ascomycetes. Fungal endophytes are especially common among the Ascomycota, representing at least five classes, dozens of families and large numbers of previously so far unknown species (Clay 1989; Gehlot et al. 2008). Most endophytes of conifer leaves are filamentous Ascomycota (Petrini 1986).

**Table 1:** Morphotypic characterization of endophytic fungi isolated from leaves of from *Cupressus torulosa* D. Don

S. no.	Code of isolate	Colony characteristics on PDA media	Slide culture	Probable endophytic fungus
1	PCTS13	Appears olivaceous brown in color	Brush-like conidiophore	*Penicillium* sp.
2	PCTS21	Appears grayish green in color	Conidiophores arose singly or in small groups, often branched, straight and flexuous	*Alternaria alternata*
3	PCTS25	Appears olive green in color but appearance of white mycelium after 10 days	The conidiophore branches at the tip. At the end of each branchlet is a cluster of spore-producing cells called phialides. A chain of spores is formed from the tip of each phialide	*Penicillium* sp.
4	KCTS14	Appears whitish gray in color	Conidiophores (annellides) produced within compact fruiting structures (acervuli or pycnidia). Spores (conidia) 4- to 5-celled, with the two or three central cells dark brown, and with two or more apical appendages or hairs; collecting in a wet mass outside the acervulus	*Pestalotiopsis* sp.
5	KCTS15	Appears gray in color	Conidiophores arose singly or in small groups, often branched, straight and flexuous	*Alternaria alternata*
6	KCTS34	Appears cottony white in color	Characterized by the presence of stolons and pigmented rhizoids, multi-spores, generally globose sporangia	Unidentified

**Table 2:** Isolated and identified endophytes from *Cupressus torulosa*, in relationship with the genus or species, and identity percentage found in the NCBI (National Center for Biotechnology Information) website

Sr. no.	Isolate code	BAB ID	Closely related Fungal sequence	% identity	Accession no.
1	PCTS13	BAB 5444	*Penicillium oxalicum*	99	KT355727
2	PCTS21	BAB 5446	*Alternaria alternata*	100	KT355729
3	PCTS25	BAB 5447	*Penicillium oxalicum*	99	KT355730
4	KCTS14	BAB 5510	*Pestalotiopsis neglecta*	99	KT355732
5	KCTS15	BAB 5445	*Alternaria alternata*	100	KT355728
6	KCTS34	BAB 5448	*Daldinia* sp.	99	KT355731

Endophytes have been intensively studied in several unexplored environments around the world (Dar et al. 2015). Endophytes were distributed in each and every plant species and were investigated for endophytic microbial components (Carroll 2004). Endophytes are chemical synthesizers inside plant. A little work in this line has been done from Garhwal, Himalayan region so for to harness the potential of hidden treasure of endophytes from indigenous plants.

Kumaran et al. (2008a) reported occurrence of *Phyllosticta spinarum* an endophyte fungus from the needles of *Cupressus* sp. which was studied as an excellent candidate for taxol production while others have earlier reported various *Pestalotiopsis* sp. from needle of *Cupressus* sp. (Maharachchikumbura et al. 2011) and from the leaves of *Pinus canariensis* (Bagyalakshmi et al. 2012) as a source of various bioactive compounds. Bisht et al. (2016) isolated and identified endophytic fungi from conifer forest plants, *C. torulosa* D.Don, which were studied with respect to production of bioactive compounds against human pathogenic bacteria. The results revealed that endophytic filamentous fungi isolates belong to the Ascomycetes group, including four different genera: *Aspergillus, Cladosporium, Alternaria* and *Curvularia*. The current study showed that *C. torulosa* is a good source of endophytic fungi, since only one type of culture medium was used for the isolation process, and this unique method allowed the isolation of a considerable number of endophytes. As no microorganism had appeared from the last washing water, so the surface disinfection method was considered efficient.

### Production and extraction of secondary metabolites

The isolates were used for the production and extraction of secondary metabolites. This isolates were extracted with two solvents, i.e., ethyl acetate and methanol.

### Antibacterial activity of crude extract by agar well diffusion method

**Fig. 1:**
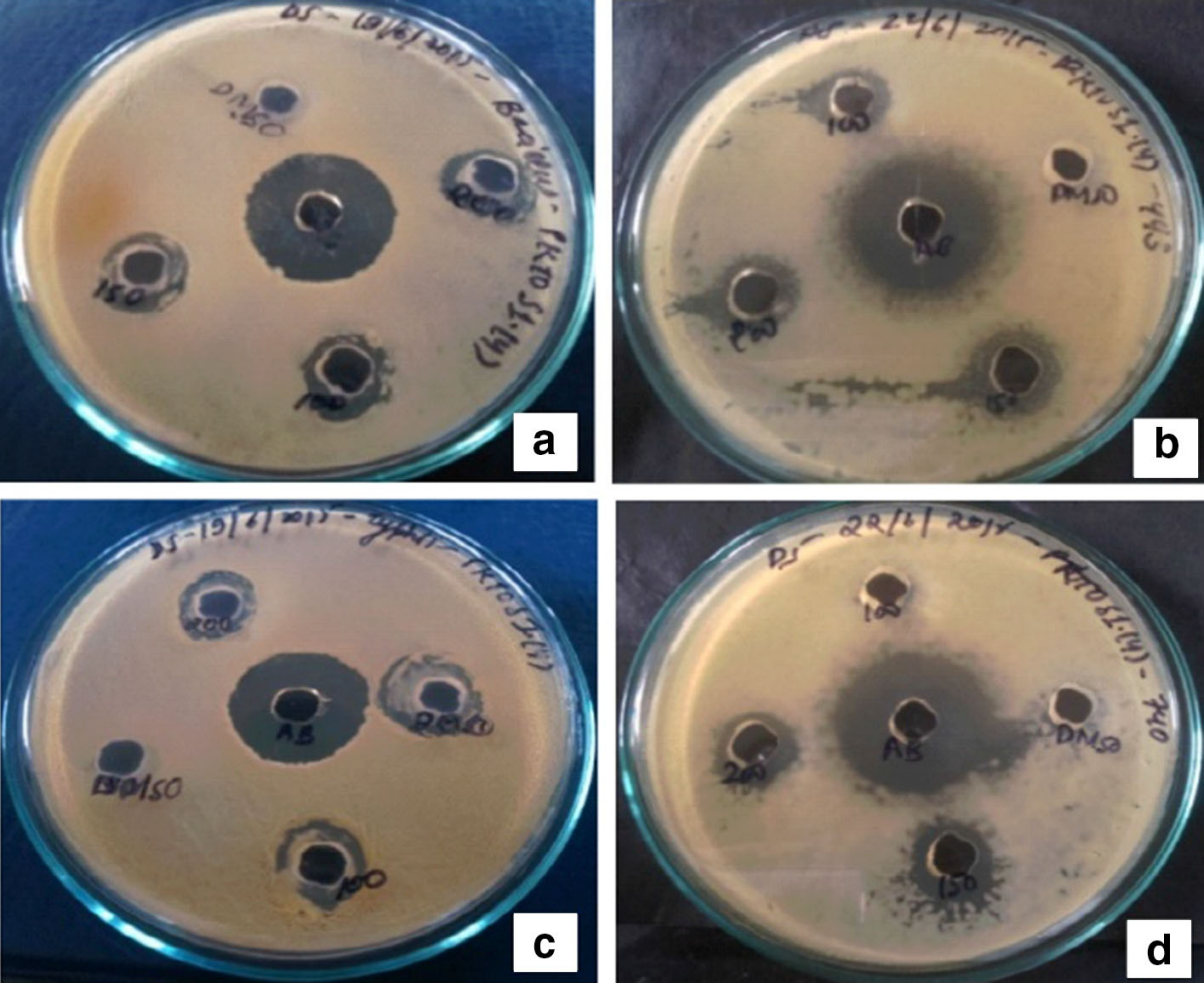
Antibacterial activity of *Pestalotiopsis neglecta* methanol extract against **a** *Bacillus subtilis*, **b** *Escherichia coli*, **c** *Salmonella typhimurium* and **d** *Staphylococcus aureus*

**Fig. 2:**
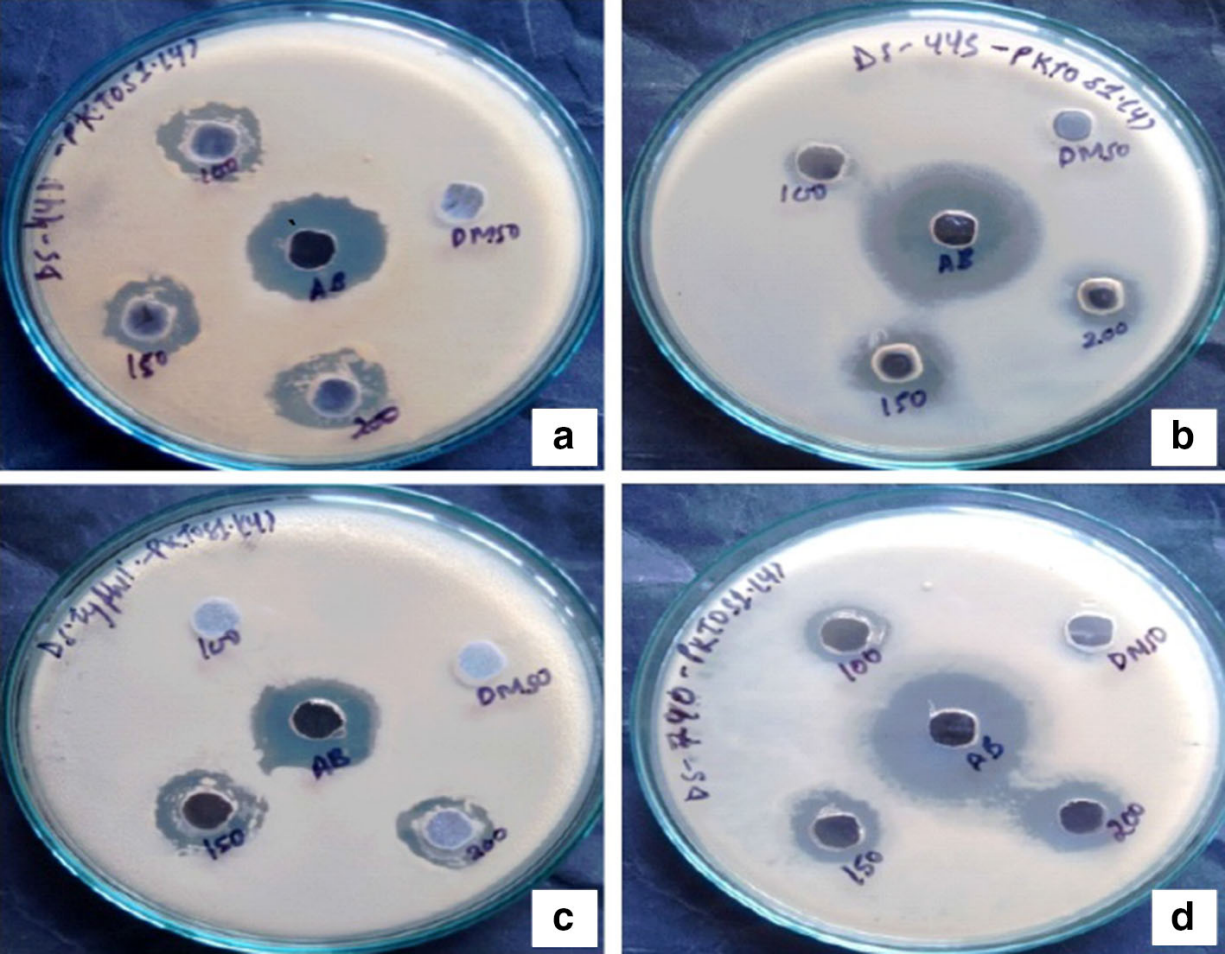
Antibacterial activity of *Pestalotiopsis neglecta* ethyl acetate extract against **a** *Bacillus subtilis*, **b** *Escherichia coli*, **c** *Salmonella typhimurium* and **d** *Staphylococcus aureus*

Fungal crude extract showed promising result by exhibiting maximum antibacterial activity against human bacterial pathogen. The screening 25 mg/mL of ethyl acetate and methanol extracts of the fungal culture was conducted using the agar diffusion method against Gram-positive and Gram-negative bacteria. All of the crude extracts of fungi isolates inhibited at least one of the microorganisms studied (data not shown). Among the tested extracts, *P. neglecta* extract inhibited the growth of the all four human pathogens; *B. subtilis, S. aureus, E. coli, S. typhimurium* and has shown broad spectrum activity which has been reported in the Table 3. The other extracts displayed significantly smaller inhibition zones when compared to *P. neglecta.* Crude extract from methanol and ethyl acetate have shown highest zone of inhibition 14.5 mm against *S. aureus* and 15 mm against *E. coli,* respectively (Figs. 1, 2). The bioactivity profiles of fungal crude extracts suggested compounds with strong and specific bioactivity. Specific bioactivity, defined as high inhibition of growth of one type of target organism with little or no activity against others, is of particular interest in drug discovery: it suggests the presence of compounds that have specific modes of action as opposed to highly toxic compounds that are often of little use as medication (Kaczorowski et al. 2011). The genus *Pestalotiopsis* has received considerable attention in recent years, because of its role as a commonly isolated endophyte which has been shown to produce a wide range of chemically novel diverse metabolites (Maharachchikumbura et al. 2011). For this reason, this fungus was chosen for further study.

**Table 3:** Antibacterial activity of fungal crude extract

Inhibition diameter zone (mm)		
Bacterial pathogens	Methanol crude extract	Ethyl acetate crude extract
*Sa*	14.5	12.0
*Ec*	14.0	15.0
*St*	11.0	12.5
*Bs*	13.0	10.0

*Bs*, *Bacillus subtilis*; *Sa*, *Staphylococcus aureus*; *Ec*, *Escherichia coli*; *St*, *Salmonella typhimurium*

Antimicrobial activity present from fungal crude extract has been conducted and proved by many studies (Garcia et al. 2012; Idris et al. 2013). Some extracts were effective against all the bacterial pathogens included in the study. These results might be attributed either to the antimicrobial potency of the extract or to the high concentration of unidentified active principle in the extracts. Other endophytic fungal extracts which showed low anti-microbial activity in the bioassay may have active compounds but probably in smaller amounts and/or the screened crude extracts could yield more potent compounds once they had undergone some purification (Idris et al. 2013). However, low activity of fungal crude extract does not indicate that this fungus does not have any activity.

Endophytic fungi isolated from spike of coniferous plant were tested with respect to their production of antimicrobial compounds against human pathogenic microorganisms such as *E. coli*, *S. aureus*, *S. typhimurium*, *Candida albicans*, *Rhizoctonia solani*, *Cladosporium herbarum* using an agar diffusion assay. The fungal crude extract did not show any activity against fungal pathogen such as *R. solani, C. herbarum*. The structural differences presented in the cellular walls of different types of bacteria and fungi (Tortora et al. 2005) are likely to affect the performance of the crude extract. This may explain why the performance of the extract was poorer in the yeast and in the Gram-negative bacterium.

Bioactive compounds isolated from endophytic fungi also displayed antimicrobial activity. Taxol, a natural product isolated from *C. cladosporioides*, showed a potent minimum inhibitory concentration against several pathogenic Gram-positive bacteria (Zhang et al. 2009), suggesting that this compound could be present in the crude extract of the fermentation of *P. neglecta*, which requires confirmation. *Pestalotiopsis* sp. from leaves of *Syzygium cumini* revealed considerable antimicrobial activity against human pathogenic bacteria viz *Staphylococcus aureus* and *Salmonella typhi* alone and in combination with commercially available antibiotics (Rahman et al. 2011). *Pestalotiopsis* *microspora* was isolated from *Taxus wallichiana* was screened using preliminary monoclonal antibody test indicate it may produce taxol (Strobel 2003). It appeared that fungi more commonly produced taxol than higher plants, and the distribution of those fungi making taxol is worldwide and not confined to endophytes of yews. Thus, it may be that taxol had its origins in certain fungi and ultimately, if there is lateral gene transfer, it may have been in the direction of the microbe to the higher plant. Similarly, Subbulakshmi et al. (2012) have reported that methanol extract of *Pestalotiopsis* sp. isolated from the *Biota orientalis* exhibited significant antibacterial and antifungal activity. Gomes Figueiredo et al. (2007) reported antibacterial activity of *Pestalotiopsis* sp. isolated from medicinal plant *Maytenus ilicifolia*, a medicinal plant from Brazil. The results revealed that the metabolites of *P. neglecta* are the potential source for the development of new antimicrobial compounds.

### Minimum inhibitory concentration of fungal crude extract

Crude extracts showing potent antibacterial activity was further examined for their MIC by a tube dilution technique against *S. aureus, B. subtilis, S. typhimurium* and *E. coli* (Table 4). Isolates of both the extracts have shown MIC ranged from 25 to 6.25 mg/mL for *S. typhimurium, S. aureus*, *E. coli* and *B. subtilis*. The methanol extract of fungal culture showed MIC of 6.25 mg/mL for *S. aureus* whereas ethyl acetate extract showed MIC of 6.25 mg/mL for *S. typhimurium* which showed its efficacy as a potent antimicrobial. The MIC of ethyl acetate extract of *Nigrospora* sp. was evaluated by tube broth dilution method was recorded as 2.5 mg/mL against *E. coli, S. aureus, C. albicans* and *Geotrichum* sp. (Pawle and Singh 2014). The methanol and ethyl acetate extracts of endophytic fungi isolated from *Indigofera suffruticosa* have shown MIC value 1.56 and 0.39 mg/mL for *S. aureus* (Santos et al. 2015). Since our study was the primary screening for the antibacterial activity of these extracts, assaying minimum inhibitory concentration (MIC) of them are suggested in order to strengthen the findings of the current study. Moreover, attempts should be made, using analytical chemistry procedures, to isolate and identify the bioactive compounds responsible for the antibacterial activity reported here.

**Table 4:** Minimum Inhibitory Concentration of the crude methanol and ethyl acetate extract of fungal isolate

Minimum inhibitory concentration (mg/mL)		
Bacterial pathogens	Methanol extract	Ethyl acetate extract
*Sa*	6.25	12.5
*Ec*	12.5	25
*St*	6.25	6.25
*Bs*	25	12.5

*Bs, Bacillus subtilis; Sa, Staphylococcus aureus; Ec*, *Escherichia coli; St, Salmonella typhimurium*

### Phytochemical screening of crude extract of endophytic fungi

Chemical analysis was carried out of fungal crude extracts to determine the presence of chemical components as a prospective source for medicinal and industrial use (Bisht et al. 2016) (Table 5). Their presence is an indicator that they can be exploited as precursors in the development and advancement of synthetic drugs. The active metabolites contain chemical groups such as phenols, flavonoids, terpenoids, alkaloids, tannins, carbohydrates and saponins. Only two phytocomponents were present in ethyl acetate extract, i.e., saponins, flavonoids, phenols and alkaloids whereas methanolic crude extract exhibited all phytocomponents except saponin. The phytochemical analyses of the ethyl acetate crude extracts of *Penicillium frequentans* have shown the presence of almost all the phytochemicals (Bhardwaj et al. 2015). The phytochemical screening of ethyl acetate extract of *Penicillium* sp. isolated from *Centella asiatica* have shown the presence of alkaloids, phenols, flavonoids, tannin and glycosides (Devi et al. 2012). The ability of an endophyte to produce some metabolites but not others has been described by (Selim et al. 2012) where different endophytes in a plant may produce different secondary metabolites hence play different functions in the plant and that the total number of metabolites in a plant extract maybe a contribution of all the endophytes that live on the plant. The production and quality of bioactive compounds from endophytic fungi depend on natural conditions of the association and the nature of the synthetic medium used (Strobel and Daisy 2003). Strategies can be developed to use these fungi for exploitation of bioactive compounds.

**Table 5:** Phytochemical screening of the methanol and ethyl acetate extracts of fungal isolate

Phytocomponents	Methanol extract	Ethyl acetate extract
Saponins	−	+
Phenols	+	+
Tannins	+	−
Terpenoids	+	−
Flavonoids	+	+
Alkaloids	+	+
Carbohydrates	+	+

### MTT cytotoxicity assay of the fungal crude extracts

**Fig. 3:**
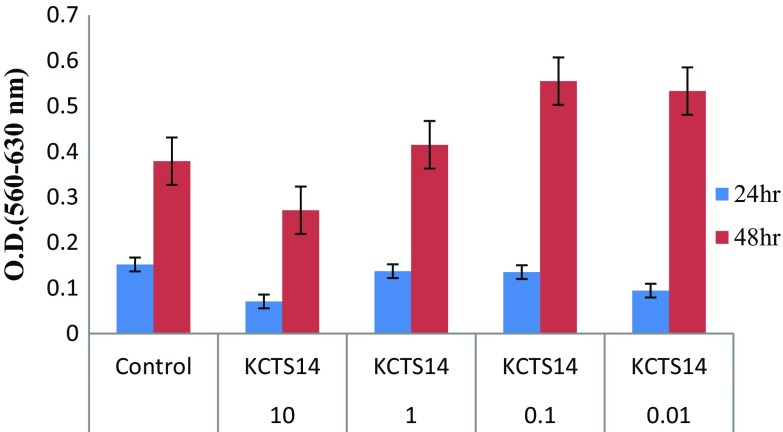
MTT cytotoxicity assay of the methanolic crude extracts of *Pestalotiopsis neglecta* (metabolic viability: Effect of KCTS14 methanol extract in HEK)

**Fig. 4:**
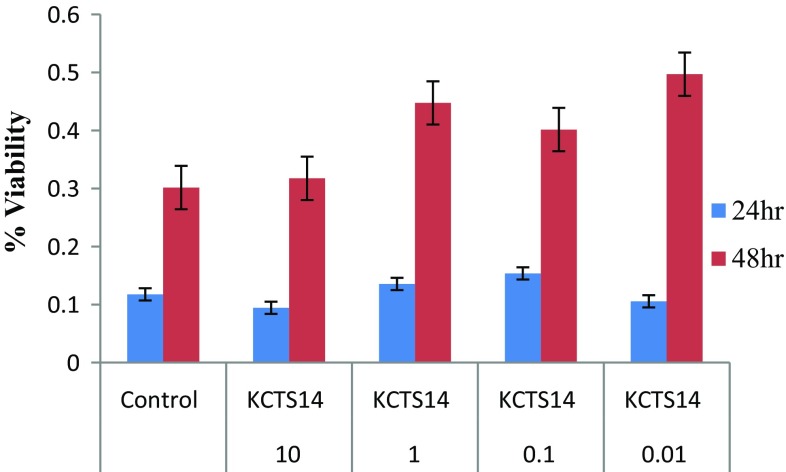
MTT cytotoxicity assay of the ethyl acetate extract crude extracts of *Pestalotiopsis neglecta* (Metabolic viability: effect of KCTS14) ethyl acetate extract in HEK)

The cytotoxic activity of methanol and ethyl acetate extracts of endophytic fungi was performed on HEK cell line to check the bioactivity of them (Figs. 3, 4). The MTT assay was carried out at different concentrations of the extracts in the cell line. The OD was measured at 560 nm with reference OD at 630 nm at 24 and 48 h. In this culture, the % viability was enhanced at 48 h and reduced at 24 h of both the crude extracts at 10 µg/mL concentration. Thus, current study showed that the effect of its secondary metabolites on the cell viability of HEK cell lines. The cells treated with the fungal extracts of concentration ranging between 125 and 500 µg/mL showed a significant decrease in the cell viability (Lakshmi and Selvi 2013). The extracts showed a high significant activity against the cancer cells. Thus, the isolate showed the potential to be used as anticancer drugs and needed to be further investigated.

Endophytic fungi extracts isolated from *Garcinia* plant were screened and 33 % of the screened extracts, showed cytotoxic activity at concentration 10 µg/mL for Vero cell line (Phongpaichit et al. 2006). Endophytic fungi isolated from *Viguiera arenaria* and *Tithonia diversifolia* was found to be cytotoxic for JURKAT cell line at concentration of 20 μg/mL (Guimaraes et al. 2008). Endophytic fungi isolated from *Bacopa monnieri* (L.) Pennell (*Scrophulariaceae*) has shown cytotoxic activity >20 μg/mL for HCT-116 cell line (Katoch et al. 2014).

Endophytic fungus BS1 was isolated from *Piper crocatum* *Ruiz* and Pav (*P. crocatum*) and have shown cytotoxic activity for WiDr and T47D cell lines with 120.38 and 37.43 µg/mL, respectively (Astuti and Nababan 2014). The fungal taxol extracted also showed a strong cytotoxic activity in the in vitro culture of human cancer cells tested in an apoptotic assay (Kumaran et al. 2008b).

### Thin layer chromatographic analysis and partial purification of bioactive compound

The fungal crude extract prepared from the cell-free culture filtrates showed strong antibacterial and cytotoxic activity. The crude extract was subjected to TLC analysis for the separation of the bioactive compounds. Two fractions designated as first and second were observed when developed in dichloromethane: methanol (90:10) on silica gel TLC plates and sprayed with ceric sulfate. These were eluted out and checked for antibacterial activity; only second fraction having Rf = 0.79 exhibited the antibacterial activity. The spot showed purplish color when sprayed with ceric sulfate. Preparative TLC was carried out to obtain sufficient crude material for further analysis.

### Detection of bioactive compounds by GC-MS analysis

**Fig. 5:**
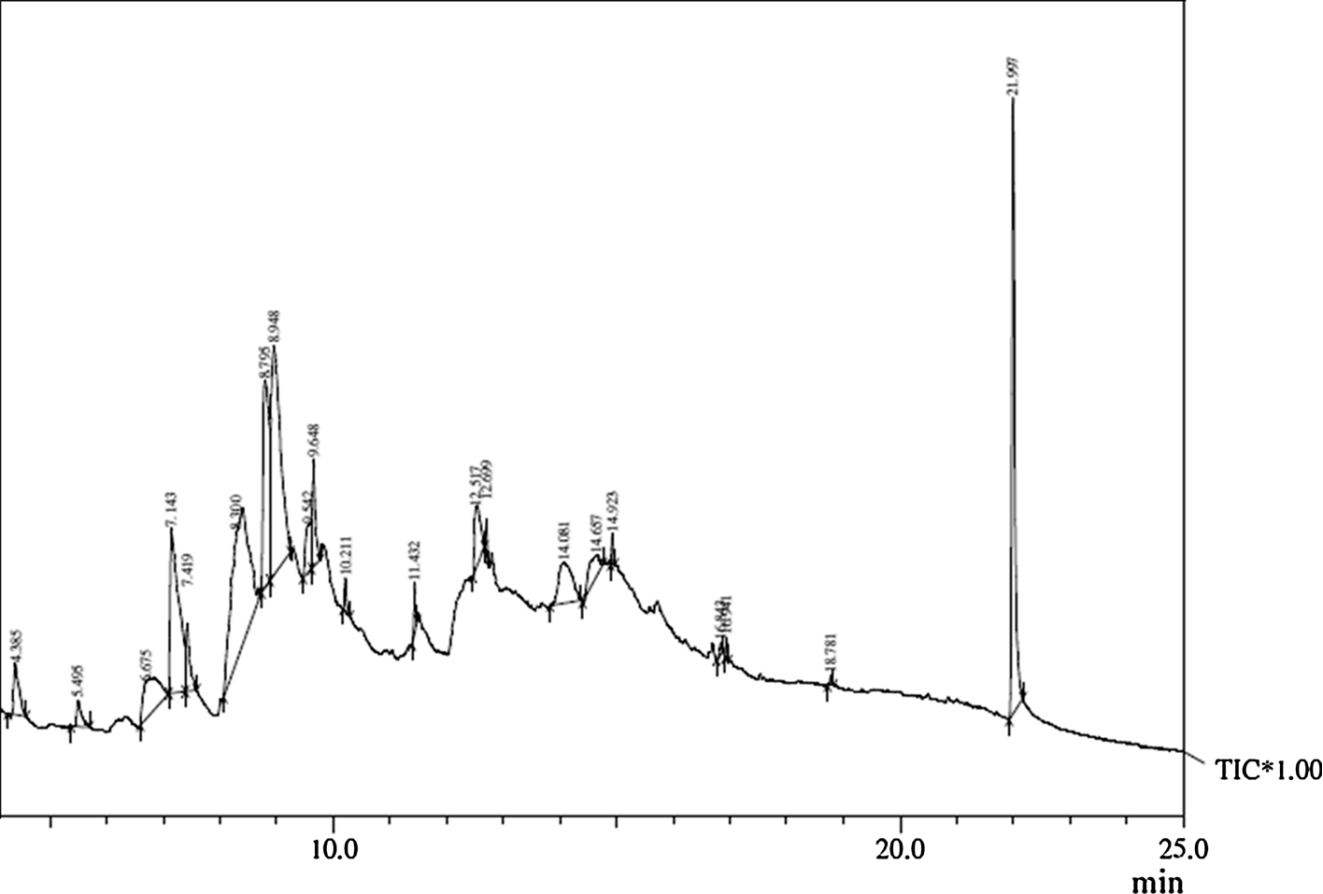
GC-MS chromatogram of KCTS14 methanol extract

The crude extract was partially purified by TLC analysis. The partially purified crude was subjected to GC–MS analysis which showed Retention time, area %, molecular formula and molecular weights of the several compounds were identified and tabulated (Table 6). The gas chromatography results of fungal crude extract reveal that major active compounds of *Pestalotiopsis* sp. BAB-5510 are nonadecane (19.74 %), 1,2,3-propanetriol, 1-acetate (17.21 %), bis(2-ethylhexyl) phthalate (14.41 %) and 4 *H*-pyran-4-one, 2,3-dihydro-3,5-dihydroxy-6-methyl- (11.62) and 5-hydroxymethylfurfural (10.09) were the compounds showed highest area %  (Fig. 5) and have antimicrobial activities and cytotoxic activity. GC–MS analysis of metabolites from endophytic fungus *Colletotrichum gloeosporioides* isolated from *Phlogacanthus thyrsiflorus* Nees have shown the presence of phenol,2,4-bis(1,1-dimethylethyl), 1-hexadecene, 1-hexadecanol, hexadecanoic acid, octadecanoic acid methyl ester and 1-nonadecane. The compounds produced by endophytic fungi could be an alternative source for human welfare (Devi and Singh 2013). GC–MS analysis of *Polycarpaea corymbosalams* also showed similar compounds such as 5-hydroxymethylfurfural (26.68) along with 2-chlorophenyl isothiocyanate (11.10) in root and n-Hexadecanoic acid (14.28) and Oleic Acid (12.94) in aerial parts were determined to be the compounds of high peak areas which mainly indulge in the anti-inflammatory properties (Sindhu and Manorama 2013). Pharmaceutical microbiology screening programs have shown that secondary metabolites can be isolated which bind to active sites of enzymes and receptors. Phthalates are reported to have antimicrobial and other pharmacological activities. Bis(ethyl hexyl) phthalate reported from *Streptomyces bangladeshiensis* showed antimicrobial activity against Gram positive bacteria and some pathogenic fungi (Al-Bari et al. 2006). First occurrence of bis-(-ethylhexyl) phthalate from *S. bangladeshiensis* and naturally occurring dioctyl phthalate showed antimicrobial activity against Gram-positive bacteria were reported very recently. The presence of various bioactive compounds (identified as phthalate esters, phthalate, alkanes, esters, alcohols, sugar, sesquiterpenoids) justifies the use of the whole plant for various ailments by traditional practitioners (Ramalakshmi and Muthuchelian 2011).

1, 2-Benzenedicarboxylic acid bis(2-ethylhexyl) phthalate has been isolated from a marine algae, *Sargassum weightii*, and apart from its plasticizing ability it was also found to have antibacterial effect on a number of bacteria (Sastry and Rao 1995; Sivakumar 2014). These obtained antibacterial compounds should then be evaluated against wider range of bacterial strains as well as in vivo, and tested for their safety and efficacy as therapeutic principles against infectious disease.

These fungi could, thus, be used to produce biofuels from cellulosics without the need for hydrolytic pretreatments. Gas chromatography-mass spectrometry-solid-phase micro-extraction (GC-S-SPME) of head space gases from an endophytic fungi, *Gliocladium* demonstrated the production of C(6)–C(19) hydrocarbons including hexane, benzene, heptane, 3,4-dimethyl hexane, 1-octene, *m*-xylene, 3-methyl nonane, dodecane, tridecane, hexadecane along with nonadecane directly from the cellulosic biomass. These fungi could potentially be developed into cost-effective biocatalysts for production of biofuels (Ahamed and Ahring 2011).

From the above findings it may be concluded that *P. neglecta* produces secondary metabolites in its culture filtrate. Thus, fungal crude extract revealed that it has the capacity to produce secondary metabolites having antimicrobial activity and cytotoxic activity. Report from this study supports the growing evidence that bioactive compounds produced by fungal endophytes may not only be involved in the host-endophyte relationship, but may also ultimately have applicability in other industries also. Endophytic fungus can be exploited for the bioactive compound. Endophytes are present in almost all plant species and have been recognized as a potential source of novel medicinal compounds. From this work, we can conclude that the endophytic fungi have wide variety of bioactive compounds. However, further research is necessary to explore the secondary metabolites of *P. neglecta* which have different biological activities.

**Table 6:** Phytocomponents identified in KCTS14 methanol extract

S. no.	RT	Name of compound	Molecular formula	MW	Area %	Activity
1	4.385	2,4-Dihydroxy-2,5-dimethyl-3(2*H*)-furan-3-one	C_6_H_8_O_4_	144	2.02	Antimicrobial
2	5.495	Pentanoic acid, 4-oxo-	C_5_H_8_O_3_	116	0.96	Antibacterial
3	6.675	Melamine	C_3_H_6_N_6_	126	4.66	–
4	7.143	4*H*-Pyran-4-one, 2,3-dihydro-3,5-dihydroxy-6-methyl-	C_6_H_12_O_2_	116	11.62	Antimicrobial, anti-inflammatory, antiproliferative
5	7.419	Dodecane	C_12_H_26_	170	2.12	Antibacterial, biofuel production
6	8.300	Nonadecane	C_3_H_6_O_3_	90	19.74	Cytotoxic effect, Antibacterial
7	8.795	5-Hydroxymethylfurfural	C_6_H_6_O_3_	126	10.09	Antimicrobial
8	8.948	1,2,3-Propanetriol, 1-acetate	C_5_H_10_O_4_	134	17.21	Antibacterial
9	9.542	Heptose	C_7_H_14_O_7_	210	2.36	–
10	9.648	Triacetin	C_9_H_14_O_6_	218	2.72	Antibacterial
11	10.211	2,3-Dihydroxypropanal	C_14_H_30_	198	0.51	Antimicrobial
12	11.432	1-Cycloheptene	C_15_H_24_	204	0.73	–
13	12.517	d-Allose	C_6_H_12_O_6_	180	2.92	Antibacterial
14	12.699	Pentadecane	C_15_H_32_	212	0.32	Antibacterial
15	14.081	1,5-Anhydrohexitol	C_6_H_12_O_5_	164	2.11	–
16	14.657	3-Deoxy-d-mannoic lactone	C_6_H_10_O_5_	162	0.39	Antimicrobial
17	14.923	Tetradecane	C_19_H_40_	268	0.20	Antimicrobial
18	16.843	1,2-Benzenedicarboxylic acid	C_22_H_34_O_4_	362	0.24	Antibacterial
19	16.941	Heneicosane	C_21_H_44_	296	0.16	Antibacterial
20	21.997	Bis(2-ethylhexyl) phthalate	C_24_H_38_O_4_	390	14.41	Antibacterial

### Phenotypic and genotypic identification of endophytic fungi

**Fig. 6:**
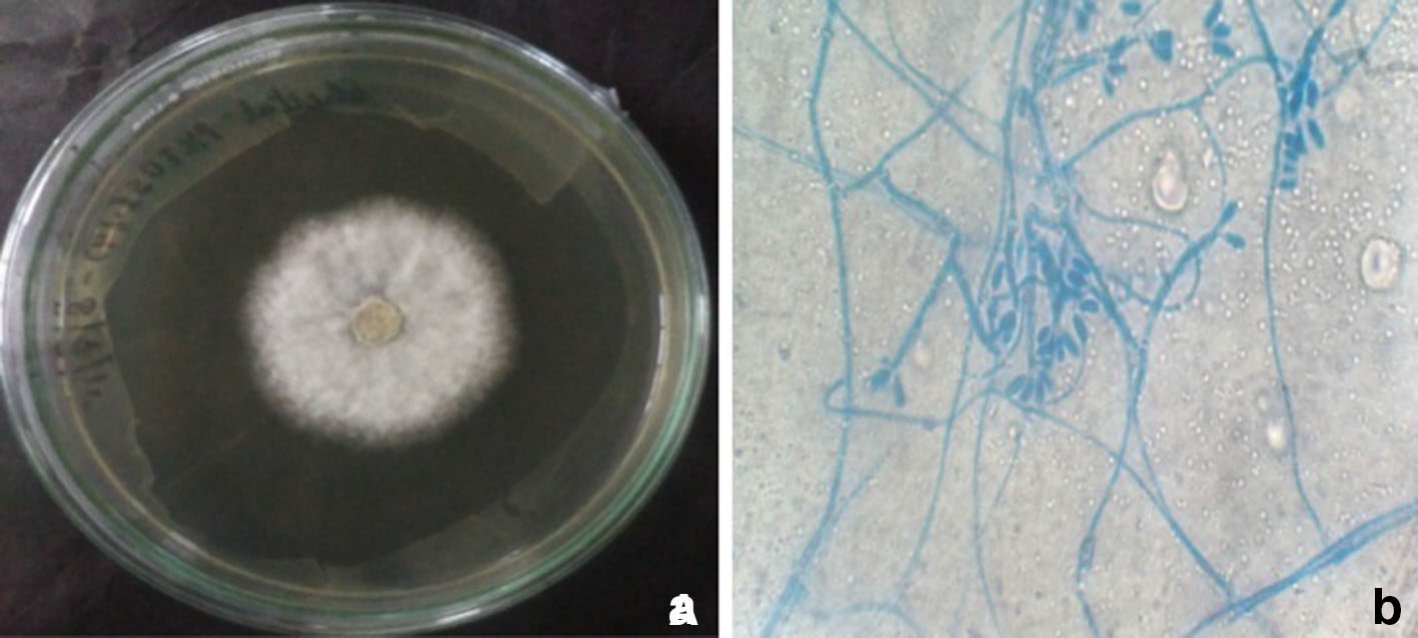
**a** Colony morphology on PDA of KCTS14 **b** Shape of conidia by staining techniques

On PDA medium, the isolated fungal culture has appeared olive green in color with threadlike mycelia with wavy margins and which change in white color on surface after a week (Fig. 6a). The slide cultures prepared from this fungus showed septate hyphae with pigmented crystals along them (Fig. 6b). In addition to the morphological characterization, genotypic methods were carried out to confirm the identification of most promising endophytic fungal strain KCTS14 isolated from the *C. torulosa* D.Don. Genotypic identification techniques were used to determine the identity of the fungus to generic level. On the basis of its 18S ribosomal RNA gene, partial sequence; internal transcribed spacer 1, 5.8S ribosomal RNA gene, and internal transcribed spacer 2, complete sequence and 28S ribosomal RNA gene, partial sequence, it can be concluded that the fungus KCTS14 belongs to the genus *P. neglecta.* The fungal sequence was submitted in National Center for Biotechnology Information with accession number KT355732 with the name *P. neglecta* BAB 5447. The percentage of similarity between the fungus and database suggests it to be a novel strain. Molecular techniques have been successfully used for identifying endophytic fungi in recent studies (Devi et al. 2012). The morphological characters having phylogenetic significance have been demonstrated and reported by Jeewon et al. (2003) and Wei et al. (2005). It was proposed that when a new *Pestalotiopsis* species is described, morphological characters should be taken into account rather than host association and molecular phylogenetic information is also necessary to prove that the taxon is unique from other known species (Jeewon et al. 2004; Wei 2004).

Endophytic fungi have a worldwide distribution from tropical forests to arctic environments and have been reported from various plants, including conifers, monocots, dicots, ferns and lycopsids (Brunner and Petrini 1992). However, this is the first report of an endophytic *P. neglecta* BAB 5447 from the Garhwal, Himalayan gymnosperm. In the current investigation, morphotypic character of fungal colony was observed in PDA plates and conidia structure by Lacto-phenol cotton blue slide staining methods. LPCB staining techniques results showed that spore release the apophyses and columella often collapse to form an umbrella-like structures which made this fungus very unusual and also difficult to identify to the species level. Therefore, genotypic methods have been used to find out the relationships of species within the genus (Brunner and Petrini 1992; Yoo and Eom 2012). More precise taxonomic identification of this fungus may require more prudent molecular techniques, expansion of fungal genomic database and further studies using several cultures of the same fungus.

## Conclusion

Endophytic fungi reside in the interior of healthy plants without causing them any damage. These fungi are of biotechnological interest; they may be used in the biological control of pests and plant diseases, and in the pharmaceutical industry. Compounds of medicinal value derived from various endophytic fungi have made immense contribution towards the betterment of human health and act as a source of inspiration for novel drug compounds. In this work, both the crude extracts of fungal isolates showed antibacterial activity against bacterial human pathogens such as *S. typhimurium, E. coli, S. aureus* and *B. subtilis.* The experiment on cytotoxic activity of endophytic fungi was performed on HEK cell line which enhanced the metabolic activity of the cells in 48 h. Both the endophytic fungal extracts also exhibited significant presence of different phytochemicals. The presence of bioactive compounds was further identified using GC-MS and shown the presence of different antimicrobial compounds. Current study concluded that fungus *P. neglecta* BAB-5510 has an ability to produce various secondary metabolites which may be used in the area of pharmacology and also as a prospective source of valuable drugs. However, isolation of individual secondary metabolite constituents and subjecting it to biological activity will definitely give fruitful results. However, further studies will need to be undertaken to ascertain fully its bioactivity, toxicity profile effect on the ecosystem and agricultural products. Insights from such research would provide alternative methods of natural product drug discovery which could be reliable, economical, and environmentally safe. The potential of these fungi is of great interest and warrants further investigation.
